# Epidemiological evidence and association of human papillomavirus with esophageal cancer in northeastern Thailand: a case–control study

**DOI:** 10.3389/fmicb.2023.1146322

**Published:** 2023-04-27

**Authors:** Ati Burassakarn, Chamsai Pientong, Panwad Tongchai, Weerayut Wongjampa, Arisara Poosari, Apiradee Udomsin, Prakasit Sa-ngiamwibool, Piti Ungareewittaya, Thitima Nutravong, Tipaya Ekalaksananan

**Affiliations:** ^1^Department of Microbiology, Faculty of Medicine, Khon Kaen University, Khon Kaen, Thailand; ^2^HPV & EBV and Carcinogenesis Research (HEC) Group, Khon Kaen University, Khon Kaen, Thailand; ^3^Ward Medic Ltd., Part., Bangkok, Thailand; ^4^Department of Pathology, Faculty of Medicine, Khon Kaen University, Khon Kaen, Thailand

**Keywords:** human papillomaviruses, esophageal neoplasms, Thailand, epidemiological characteristics, meta-analysis

## Abstract

Recently, epidemiological evidence of high-risk human papillomavirus (hrHPV) and its association with the increasing risk of esophageal cancer (EC) have been described. However, the involvement of such a virus in the pathogenesis of EC is still inconclusive in the literature. Therefore, our objective was to clarify the epidemiology of HPV infections in primarily diagnosed EC cases and validate this correlation with hospital-based control patients using a retrospective study with a case–control model. Here, we reported that the overall prevalence of HPV DNA was statistically associated with an increased risk of EC (OR, 3.3; 95% CI, 2.5–4.3). Interestingly, a history of gastroesophageal reflux disease (GERD) was constituted and significantly associated with HPV prevalence (adjusted OR, 4.6; 95% CI, 2.2–9.5). Furthermore, our meta-analysis in public databases also indicated that the combined OR and 95% CI between HPV infection and EC risk were 3.31 and 2.53–4.34, respectively, with significant heterogeneity (*I*_2_ = 78%). Variations in the geographic study, tissue type, and detection method remain potential predictors of heterogeneity. In addition, publication bias and sensitivity analysis were not observed, and the results exhibited stable outcomes. Collectively, we specify the recent epidemiological evidence in a validation of the distributed HPV, which might be statistically associated with an increased risk of EC. However, additional high-quality studies with larger sample sizes are needed to further verify the link between HPV and EC.

## 1. Introduction

Esophageal cancer (EC) is the tenth most common cancer worldwide with a poor prognosis in clinical practice (Allemani et al., 2018). According to the report by the IARC, 3.1% accounts for new cases (604,100 cases of all cancers), and 5.5% accounts for impermanence (544,076 deaths in total) in 2020 (Sung et al., 2021). Based on the principle of microscopic pathology, EC can be classified into two main histologic subtypes: adenocarcinoma (EAC, 12%) and squamous cell carcinoma (ESCC, 88%; Arnold et al., 2015). It has well-defined distinctive geographical patterns and risk factors for these diseases. Although EAC is associated with Barrett’s esophagus, smoking, tobacco use, a history of gastro-esophageal reflux disease (GERD), and obesity, ESCC has been associated with alcohol consumption and smoking. In general, almost 80% of the total cases occur in the regions of developing countries (El-Serag et al., 2014; Rustgi and El-Serag, 2014; Coleman et al., 2018; Morgan et al., 2022). However, EAC has commonly been found in developed counties—particularly in Westerners due to changes in risk factor contours in these circumstances (Rumgay et al., 2021).

Human papillomaviruses (HPVs) are a type of ubiquitous double-stranded circular DNA with non-enveloped, small viruses that have been classified as the family, *Papillomaviridae*. Currently, more than 150 HPV genotypes have been identified and categorized into cutaneous and mucosal subgroups. It is well known that mucosal HPV types, specifically HPV16, 18, 31, and 33, are also recognized as high-risk HPV (hrHPV) and are the causative agents of several types of cancer in humans (Gheit, 2019). Of these, HPV16 is the most potent oncogenic genotype of the hrHPVs subgroup, according to previous epidemiologic studies (Tsikouras et al., 2016; Petrelli et al., 2021). In addition to the cancer of the genital tract (i.e., cervix, vagina, vulva, anus, and penis) and a subset of head and neck carcinomas (HNSCCs), particularly oropharyngeal cancers (OPSCCs), the pieces of evidence for the association between hrHPV infection and the increasing risk of esophageal cancers (ECs) have recently been described (Rajendra et al., 2020). In the early 1980s, the hypothesis of HPV-linked genital tract lesions and ESCC was first introduced by Syrjanen et al. (1982). The works from a pioneer group showed that the average frequency of HPVs was 29.0% (range: 15%–78%) and found the distinct prevalence of any HPV type in ESCCs (Syrjänen, 2002), while the studies using the larger scale case–control model suggested that the range rate of HPV infection was between 0% and 100% (Ludmir et al., 2015). These reports also indicated that HPV prevalence was reasonably elevated in high-burden regions of the world’s EC (Petrelli et al., 2021). For example, the HPV infection rate (32.8%–63.6%) in EC cases from countries with extraordinary rates of overall EC incidence, particularly Northern China and Iran, was significantly higher than in countries with a lower incidence of EC. On the contrary, a lower percentage of HPV-infected EC was observed in regions with lower EC incidence such as Europe (15.6%) and the United States (16.6%; Hardefeldt et al., 2014). Furthermore, Rajendra et al. (2013) also exhibited the first strong evidence for the relationship between transcriptionally active hrHPV and EAC. Interestingly, a positive trend correlation between HPV16 and ESCCs as well as HPV18 and EACs was also presented by these epidemiological reports (Petrelli et al., 2021; Li et al., 2022). However, the true prevalence and contribution of HPV in EC remain to be passionately deliberated, conferring the partially unknown and capricious findings among several studies from various geographical settings (Kunzmann et al., 2017; Hošnjak and Poljak, 2018).

Esophageal cancer (EC) is one of leading cancers in Thailand (ranked 16th). Based on world ASR per 100,000, the incidence and mortality of this malignancy in Thailand were 2.9 and 2.7, respectively (Sung et al., 2021). It appears to be a considerable clinical and public health issue in Thailand. Despite well-known environmental factors, infectious agents have also affected more than one-quarter of the considerable cancer nationwide. Moreover, Thailand is situated in the high-risk flight path of esophageal cancer in Southeast Asia, where patients often do not describe the viral-related risk factors, reflecting the limitation of identified causative agents (Sunpaweravong, 2010). Even though the potentially pivotal role of HPVs, particularly high-risk types involved in the biological consequence and malignant transformation of the esophageal mucosa have been suggested and reported by the former pieces of evidence (Kunzmann et al., 2017; Hošnjak and Poljak, 2018). However, an association between HPV infection and the risk of esophageal neoplasms is not conclusive in the accumulated literature. Regarding the meta-analyzed findings, HPV infection could be an etiology that plays an important role in esophageal carcinogenesis, especially in the squamous cell carcinoma (SCC) subtype in some regions, indicating the variation in its geographical incidence, which was affected by exposure to dietary, cultural, environmental, and any specific factors. Therefore, these findings have led us to not make any decisive statements about the association between HPV infection and esophageal malignancies compared to other types of cancer, particularly oropharyngeal cancer. To this end, in this study, our objective was to highlight the frequency of HPV infections in the primary diagnosis of EC cases and validate this correlation with hospitalized control patients using a retrospective study model, accompanied by meta-analyses of published information in public databases. Corroborating this information might amend our comprehension of HPVs status in EC and provide some pieces of HPV-associated EC evidence for clinical management, improving disease outcome and prognosis.

## 2. Materials and methods

### 2.1. Ethics statement

This study was registered and approved by the Ethics Committee of the Khon Kaen University for Human Research (Reference no. HE621269). Informed consent was obtained from all volunteers who were participants in this study.

### 2.2. Study site

Based on the prevalence of EC cases in Thailand (Cancer in Thailand, 2022), this study was carried out at Srinagarind Hospital, a university medical teaching hospital, Faculty of Medicine, Khon Kaen University, Khon Kaen, Thailand (16°28′6″N 102°49′48″ E).

### 2.3. Study design and research participants

According to our previous study (Poosari et al., 2021, 2022), we dependably conducted a retrospective study with a total of 105 sample volunteers in each patient case who was diagnosed primarily with EC and the control calibrator from hospital subjects. All cases with multiple primary-diagnosed malignancies or other cancer origins, or those with a history of cancer treatment, were excluded. The healthy dependents who endured the examination of their physical body and clinical manifestation without a history of gastric or esophageal malignancy by the hospital physician during the period of case recruitment were registered in this study as the calibrated control group. Clinico-epidemiological characteristic information on each subject including sex, age, physical height, body weight, smoking status and frequency, alcohol consumption, history of GERD, oral hygiene practices, and a family history of cancer was achieved by the structured interview questionnaire.

### 2.4. Tissue sample collections and processing

Formalin-fixed paraffin-embedded (FFPE) tissue samples of both EC patients and control were retrospectively retrieved from the Department of Pathology, Faculty of Medicine, Khon Kaen University, between 2015 and 2018. Based on the guidance from the International Classification of Diseases in Oncology, 3rd edition (ICD-O-3; codes: C15.3-C16.0; Fritz et al., 2013), the samples were histologically reviewed and confirmed by two independent pathologists using a histopathological report and a standard hematoxylin–eosin (H&E) staining technique (H&E), respectively. Before a step of HPV detection, 5 μm of thick serial sections were cut from each FFPE sample and placed in a sterile microcentrifuge. The tube can be stored at room temperature until used.

### 2.5. Determination of HPV infection using the HPV direct flow CHIP technique

As previously described by Herraez-Hernandez et al. (2013), HPV detection and genotyping in the FFPE tissue sample were carried out using the HPV Direct Flow CHIP system. In brief, the mixture of 60 μL Lysis Buffer (Master Diagnóstica, Granada, Spain) and 1.5 μL of DNA Release (Master Diagnóstica, Granada, Spain) was used to digest the three serially sectioned tissues at 60°C for 30 min, followed by inactivation at 98°C for 10 min. The extracts were then collected. In total, 4 μL of the extracts were added to a 36 μL PCR mix supplied kit (Master Diagnóstica, Granada, Spain). The product was amplified by a T 100™ Thermal Cycler (Bio-Rad, Hercules, CA, United States) using the cycling conditions at 25°C for 10 min; 94°C for 3 min; 15 cycles of denaturation at 94°C for 30 s, annealing at 42°C for 30 s, and elongation at 72°C for 30 s; 35 cycles of denaturation at 94°C for 30 s, annealing at 60°C for 30 s, and elongation at 72°C for 30 s; and final elongation at 72°C for 5 min. The PCR products were stored at −20°C until use.

For detection and genotyping, the biotinylated amplicons were first denatured at 95°C for 5 min, followed by cooling in an ice bath for 2 min. The hybridization was carried out in sets of 12 samples using the hybridSpot 12 (HS12) platforms (Master Diagnóstica, Granada, Spain), according to the manufacturer. The DNA target is primarily crossed and binds to HPV CHIP membranes containing immobilized complementary probes that are specified in the beta-globin gene, consensus, and genotype-specific sequences of HPV. The colorimetric assessment was achieved by applying NBT-BCIP substrates that detect alkaline phosphatase activity, developing insoluble purple precipitates.

### 2.6. Analysis of risk factors and hrHPV responsiveness in EC

The basic information on gender, age, and the well-known risk factors of EC, including status and frequency of smoking, alcohol consumption, GERD history, and a family history of cancer, was selected and analyzed. The posed data of these demographic characteristics and risk factors might be associated with infections of hrHPV. Furthermore, the answers of all participants seem to reflect their level of knowledge and overall attitudes toward the correlation between EC and potential environmental risk factors. In this study, each participant directly answered or read all the questions by trained personnel.

### 2.7. Systematic review and meta-analysis of HPV-associated esophageal malignancies

The depth and evenness of the existence of HPV in EC imply a hypothetically vital role for HPV infection in the oncogenesis of esophagus origin. Therefore, we conducted a meta-analysis to assess the association between them.

#### 2.7.1. Strategy for searching the pieces of literature

The four main public databases including PubMed, SCOPUS, Excerpta Medica database (EMBASE), and Cochrane Library were restricted in English until 31 September 2022 using the combination of medical subject headings (MeSH; “esophageal neoplasms”) OR the following keywords in [All Fields] of (“esophagus” AND “cancer”) OR (“esophageal cancer”) OR (“esophagus” AND “neoplasm”) OR (“ESCC”) OR (“EC”) AND (“human papillomavirus”) OR (“HPV”; Petrelli et al., 2021; Geng et al., 2022). In addition, the reference lists from previous systematic reviews and recovered articles were also checked.

#### 2.7.2. Selection of the retrieved articles

The reports had to meet the following criteria to be included: (1) the studies that assessed the infection of HPV in tissue samples from healthy participants (control group) and eventually compared them with the histologically approved EC with at least 10 cases, (2) the studies with inline a case–control model were eligible, (3) any place, race, sex, age, and cancer stage was no limitation. Patients with cancer treatments were not eligible for this analysis, (4) the articles provided sufficient data to observe and/or calculate odds ratio (OR) and relative risk (RR), with a 95% confidence interval (95% CI), and (5) the most recent reports on the same population included a larger number of participants. Furthermore, studies that were implemented with preclinical models (i.e., *in vitro* or animal studies) or other types of publication including case reports, personal opinions, conference abstracts, letters, book chapters, and reviews were excluded. The notion of data overlap or duplicate articles was also not eligible for extraction and quality assessments.

### 2.8. Extraction and quality assessments of the retrieved data

To characterize potentially eligible reports, a recent study was conducted according to the Preferred Reporting Items for Systematic Reviews and Meta-Analysis (PRISMA) guidelines (Page et al., 2020). Titles, abstracts, and the following information from the full main text of the retrieved articles were obtained: first author, year of publication, studied location/country, design of study model, sample number, demographic characterization of the participants, adjusted variables (i.e., confounding factors), and risk estimates (e.g., OR, RR with 95% CI) were independently investigated and assessed by three reviewers (AB, PT, and WW) accompanied with the pre-delineated criteria. The discrepancies or inconclusive results were consulted and resolved by the fourth reviewer (AP). Furthermore, the bias risk of all included studies was separately validated by the three reviewers using the Newcastle–Ottawa Scale (NOS) guidelines (Wells et al., 2014). Of these, 9 points are the maximum NOS score, and the high-quality articles had to be considered as scored 6 (Wells et al., 2014; Geng et al., 2022). All analyses were performed on previously published articles; thus, both ethical documents and the consent forms of the patients were not mandatory.

### 2.9. Statistical analysis

For our case–control study, descriptive statistics were used to synopsize the demographic characteristics of the participants. The categorical records and the continuous data were represented in frequencies and percentages and the numbers by mean ± standard deviation (SD), median, and minimum and maximum ranges, respectively. Bivariate analysis was performed using simple logistic regression to verify the association between EC and potential independent risk factors. While the crude odds ratio (OR_c_) with 95% CI was calculated for the bivariate analysis with uncontrolled confounders, the adjusted odds ratio (OR_adj_) with 95% CI was estimated by multivariate unconditional logistic regression for the controlling confounders (OR_c_; *p* < 0.25 with EC association; Poosari et al., 2021). The backward stepwise elimination strategy was used to construct the simple and interpretable regression model. Consequently, the goodness-of-fit of the final model was evaluated by a likelihood ratio test. All HPV detection experiments were carried out in triplicate. The two-tailed test analyzed the statistical results, and a *p*-value of <0.05 was considered statistically significant. This analysis was performed using SPSS software version 14.0 (SPSS Inc., Chicago, IL, United States) and GraphPad Prism version 8.0 (GraphPad Software, Inc., San Diego, CA, United States).

For the meta-analysis, the pooled odds ratios (ORs) with the corresponding 95% CI were calculated from the total of EC cases and controls. Forest plots were visually constructed to determine the study’s pooled effects and specificity. Using Cochran’s Q test, the *I* statistic was performed to evaluate heterogeneity as low (*I*_2_ > 25%), moderate (*I*_2_ > 50%), and high (*I*_2_ > 75%) levels in the retrieved reports. Further analysis of heterogeneity was performed leave-one-out with a random effect model when significant articles (*p* < 0.01 or *I*_2_ > 50%) were observed (Higgins and Thompson, 2002; Higgins et al., 2003). Or else, the fixed effect model was subsequently applied (DerSimonian and Laird, 1986). After omitting heterogeneous studies, the sensitivity analysis was conducted, accompanied by the stigmatization of the new value of the OR. The possible potential effects on heterogeneity were determined by subgroup analyses based on crucial study traits including study sites, number of samples, detection methods/markers, and others. To assess the impacts of the aforementioned factors on the association between HPV infection and EC risk, the meta-regression analysis was also then applied. The potential bias of publication was examined by Begg’s and Egger’s tests (Begg and Mazumdar, 1994; Egger et al., 1997). Visual inspection of the asymmetry of a funnel plot was constructed to consider bias. All analyses of the statistics were performed with the Review Manager (RevMan) software version 5.4.1.,^1^ SPSS software version 14.0 (SPSS Inc., Chicago, IL, United States), and GraphPad Prism version 8.0 (GraphPad Software, Inc., San Diego, CA, United States). If there is no specific mention, the statistical results were analyzed with the two-sided test, and a *p*-value of <0.05 was considered statistically significant.

## 3. Results

### 3.1. Clinico-demographic characteristics of patient cases and control participants

To determine the association between HPV infections and increased risk of ECs, we conducted a recent study in a case–control-dependent manner. A total of 100 EC patients and 105 hospital-based volunteers (control) group were enrolled. Of the total cases, the median age of the EC patients at diagnosis was 59.5 years (range, 38–78 years old), and male patients (64.0%) had a higher prevalence of EC than those of female (36%) patients. This group’s most common histopathological diagnosis was squamous cell carcinoma (SCC; 72.0%) and tissue grading with poor differentiation (66.0%). Stage IV (66.0%) of the TNM classification was the highest frequency in registered patients. Interestingly, the difference in lifestyle behaviors between cases and controls was statistically observed. Cases had a higher prevalence of smoking (72.0% vs. 26.7%) and alcohol consumption (78.0% vs. 41.0%) than controls. Moreover, the cases also significantly diverged from the controls in a history of gastroesophageal reflux disease (GERD; 79.0% vs. 37.1%) and a family history of cancer (FHC; 73.0% vs. 41.9%). The presence of HPV DNA was subsequently found in a total of 45 (45.0%) EC cases, while 23 (21.9%) paradigms of the controls were correspondingly achieved for such viral DNA. These demographic characteristics of all participants are described in Table 1.

**Table 1:** Clinico-demographic profile of esophageal cancer patients and control participants.

Characteristics	Cases	Control	*p*-value^a^
	*N* = 100; *n* (%)	*N* = 105; *n* (%)	
Gender			
Male	64 (64.0)	51 (48.6)	0.026
Female	36 (36.0)	54 (51.4)	
Median of participant’s age; year (range)	59.5 (38–78)	54 (2–86)	0.005^b^
Smoking status			
No	28 (28.0)	77 (73.3)	<0.001
Yes	72 (72.0)	28 (26.7)	
Alcohol consumption			
No	22 (22.0)	62 (59.0)	<0.001
Yes	78 (78.0)	43 (41.0)	
History of gastroesophageal reflux disease (GERD)			
No	21 (21.0)	66 (62.9)	<0.001
Yes	79 (79.0)	39 (37.1)	
Family history of cancers (FHC)			
No	27 (27.0)	61 (58.1)	<0.001
Yes	73 (73.0)	44 (41.9)	
Histopathological diagnosis			
Squamous cell carcinoma (ESCC)	72 (72.0)	–	ND
Adenocarcinoma (EAC)	28 (28.0)	–	
Histopathological grade			
Poor differentiation	66 (66.0)	–	ND
Moderate or Well differentiation	30 (30.0)	–	
Indetermination	4 (4.0)	–	
TNM stage			
Stage I, II	9 (9.0)	–	ND
Stage III	24 (24.0)	–	
Stage IV	66 (66.0)	–	
HPV detection			
Negative	55 (55.0)	82 (78.1)	<0.001
Positive	45 (45.0)	23 (21.9)	

ND, not determined the differences in *p*-values.

a *p*-values derived from the Chi-square test.

b The Kruskal–Wallis test was applied to determine the differences in *p*-values between cases and controls.

### 3.2. Frequency and distribution of HPV DNA in biological tissue samples

**Figure 1:**
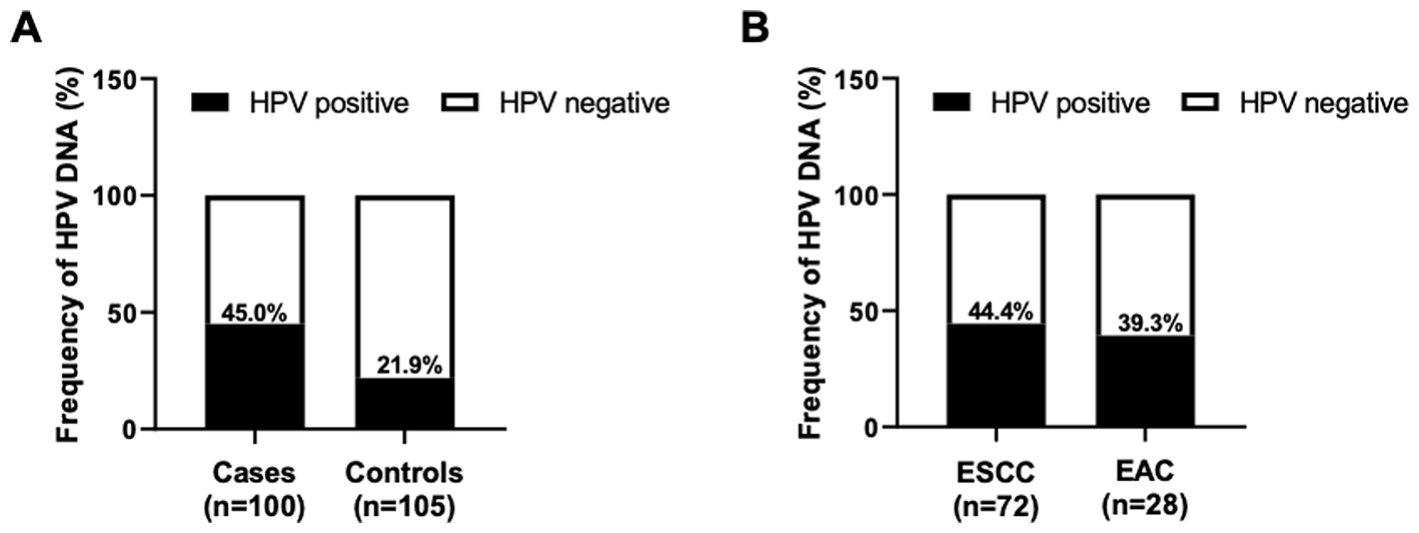
Frequency of HPV DNA in EC tissue samples. The column graph demonstrated the frequency of HPV DNA detected by the HPV Direct Flow CHIP technique in the FFPE specimens collected from EC cases and control subjects **(A)**. The infection rate of HPV in different histology subtypes of EC was also illustrated **(B)**.

**Figure 2:**
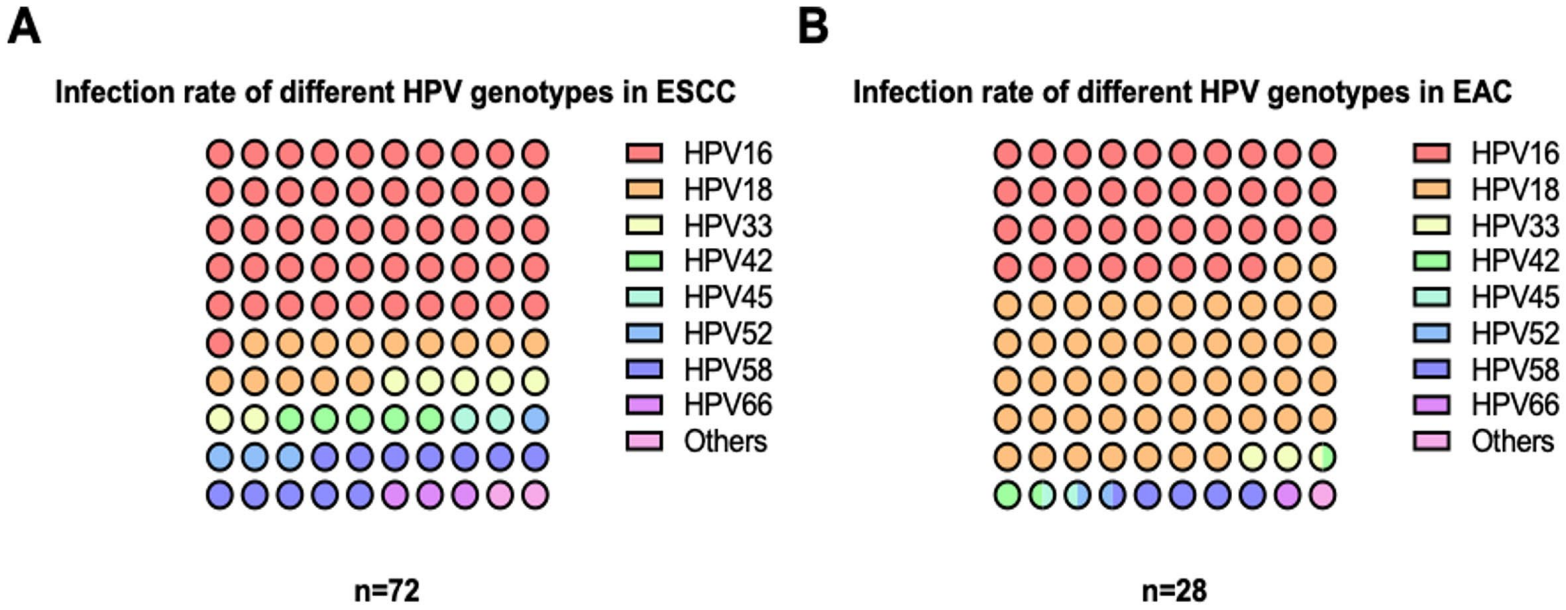
Distribution of HPV genotypes in EC tissues. The dissemination of circulated HPV genotypes in each EC subtype indicated that HPV16, HPV18, and HPV58 were the top three predominated genotypes in ESCC **(A)** and EAC **(B)**, respectively.

Although the association between HPV infection and EC risk has been postulated, the viral contribution to the pathogenesis of EC is partially unknown and capricious in the literature. To this end, the detection of HPV was subsequently performed using the HPV Direct Flow CHIP, targeting the viral genome. DNA from tissue specimens of all patient cases and control subjects was available for testing as it exhibited β-globin-positive results (Table 2). Of the 72 samples of ESCC, 32 cases (44.4%) were labeled HPV DNA-positive. Additionally, our detection illustrated a total of 11 HPV-positive paradigms (39.3%) of the 28 types of EAC tissue. On the contrary, we also accounted for the viral DNA in 23 of 105 (21.9%) of the control group (Table 2; Figure 1). Fascinatingly, the presence of HPV DNA was statistically associated with an increased risk of total EC cases (OR_c_, 2.9; 95% CI, 1.6–5.4; *p* < 0.001) and ESCC subtypes (OR_c_, 3.2; 95% CI, 1.2–6.1; *p* < 0.001). However, our result revealed that there was no statistical difference in the association between the existing viral DNA and the EAC (OR_c_, 2.3; 95% CI, 0.9–5.6; *p* < 0.0651; Table 2). Similarly, of the positive samples, HPV16 (51.0%) was the most common genotype found in ESCC, while HPV18 (49.2%) was the highest positive rate in EAC. However, HPV58 was also found in both subtypes of EC with the highest positive rate (approximately 10%–14%), as illustrated in Figure 2. Taken together, recent information suggested that the contribution of HPV infection could particularly be HPV16, HPV18, and HPV58 to the increased risk of ECs.

**Table 2:** Occurrence and involvement of HPVs in esophageal cancer (EC) risk.

Clinical specimen			Human papillomaviruses (HPVs)		
Participant	Type of specimen^a^	No. of available specimens^b^	Prevalence *n*, (%)	OR_c_ (95% CI)	*p*-value
ESCC	Tissue	72	34 (44.4)	3.2 (1.2–6.1)	<0.001
EAC	Tissue	28	11 (39.3)	2.3 (0.9–5.6)	0.0651
Total		100	45 (45.0)	2.9 (1.6–5.4)	<0.001
Control	Tissue	105	23 (21.9)	1.0 (reference)	ND
Total		105	23 (21.9)	1.0 (reference)	ND

OR_c_, crude odd ratio; CI, confidence interval; ND, not determined the differences in *p*-values; ESCC, squamous cell carcinoma; EAC, adenocarcinoma.

a Formalin-fixed paraffin-embedded tissue.

b β-globin positive.

### 3.3. HPV infection could be an independent risk factor for EC

It is well known that the pieces of evidence for the involvement of various living behavior-related (environmental) factors and/or intrinsic factors on EC pathogenesis have been suggested (Morgan et al., 2022). Therefore, we consequently hypothesized whether these potential factors might be related to the risk of EC, together with HPV infection. Of those factors, a total of six potential risks, including age, gender, the status of smoking, alcohol consumption, history of GERD, and FHC, were acknowledged in the current study. Our univariate logistic analysis indicated that smoking (OR_c_, 7.1; 95% CI, 3.8–13.1; *p* < 0.001), alcohol consumption (OR_c_, 5.1; 95% CI, 2.8–9.4; *p* < 0.001), history of GERD (OR_c_, 6.4; 95% CI, 3.4–11.9; *p* < 0.001), and FHC (OR_c_, 3.7; 95% CI, 2.1–6.7; *p* < 0.001) were statistically associated with an increased risk of EC, synergizing with HPV infection (Table 3). Regardless of the promised risk factors, only smoking cases (OR_adj_, 4.2; 95% CI, 1.8–9.8; *p* < 0.001) and patients who had a history of GERD (OR_adj_, 4.6; 95% CI, 2.2–9.5; *p* < 0.001) were statistically dependently correlated with HPV infection (OR_adj_, 3.7; 95% CI, 1.7–7.8; *p* < 0.001), in the EC risk context by the analysis of multivariate logistic regression (Table 3). However, our evaluations have shown that smoking (OR_adj_, 1.2; 95% CI, 0.5–2.8; *p* = 0.749) was not statistically associated with HPV infection in patients with EC (Table 4). Compared with patients with a history of GERD, the frequency of HPV DNA was fashionably decreasing and was not statistically associated with HPV infection (OR_adj_, 0.9; 95% CI, 0.3–2.3; *p* < 0.747; Table 4). Collectively, our contemporary evidence implied that infection with HPV could be an independent risk factor for ECs, supporting the etiologically viral agent of such disease.

**Table 3:** The association between various clinicopathologic risk factors and ECs.

Characteristics	Cases	Control	OR_c_ (95% CI)	*p*-value	OR_adj_ (95% CI)	*p*-value^a^
	*N* = 100; *n* (%)	*N* = 105; *n* (%)				
Gender						
Male	64 (64.0)	51 (48.6)	1.9 (1.1–3.3)	0.0267	ND	ND
Female	36 (36.0)	54 (51.4)	1.0 (reference)			
Age						
≥60	50 (50.0)	38 (36.2)	1.7 (1.0–3.1)	0.047	ND	ND
<60	50 (50.0)	67 (63.8)	1.0 (reference)			
Smoking status						
Yes	72 (72.0)	28 (26.7)	7.1 (3.8–13.1)	<0.001	4.2 (1.8–9.8)	<0.001
No	28 (28.0)	77 (73.3)	1.0 (reference)		1.0 (reference)	
Alcohol consumption						
Yes	78 (78.0)	43 (41.0)	5.1 (2.8–9.4)	<0.001	1.2 (0.5–3.0)	0.619
No	22 (22.0)	62 (59.0)	1.0 (reference)		1.0 (reference)	
History of GERD						
Yes	79 (79.0)	39 (37.1)	6.4 (3.4–11.9)	<0.001	4.6 (2.2–9.5)	<0.001
No	21 (21.0)	66 (62.9)	1.0 (reference)		1.0 (reference)	
FHC						
Yes	73 (73.0)	44 (41.9)	3.7 (2.1–6.7)	<0.001	1.8 (0.9–3.6)	0.111
No	27 (27.0)	61 (58.1)	1.0 (reference)		1.0 (reference)	
HPVs detection						
Yes	45 (45.0)	23 (21.9)	2.9 (1.6–5.4)	<0.001	3.7 (1.7–7.8)	<0.001
No	55 (55.0)	82 (78.1)	1.0 (reference)		1.0 (reference)	

HPVs, human papillomaviruses; OR_c_, crude odd ratio; OR_adj_, adjusted odd ratio; CI, confidence interval; GERD, gastroesophageal reflux disease; FHC, Family history of cancers; ND, not determined.

a *p*-value was obtained from a multiple logistic regression model with all conditional adjusted variables.

**Table 4:** The correlation of potential clinicopathologic findings and HPVs status in ECs.

Characteristics	ECs (N = 100)		OR_adj_ (95% CI)	*p*-value^a^
	HPVs positive; *n* (%)	HPVs negative; *n* (%)		
Smoking status				
Yes	33 (73.3)	39 (70.9)	1.2 (0.5–2.8)	0.749
No	12 (26.7)	16 (29.1)	1.0 (reference)	
History of GERD				
Yes	35 (77.8)	44 (80.0)	0.9 (0.3 – 2.3)	0.747
No	10 (22.2)	11 (20.0)	1.0 (reference)	

HPVs, human papillomaviruses; OR_adj_, adjusted odd ratio; CI, confidence interval; GERD, gastroesophageal reflux disease.

a *p*-value was obtained from a multiple logistic regression model with all conditional adjusted variables.

### 3.4. The frequency of HPV DNA steadily increased in the EC tissue samples throughout the time collection

**Figure 3:**
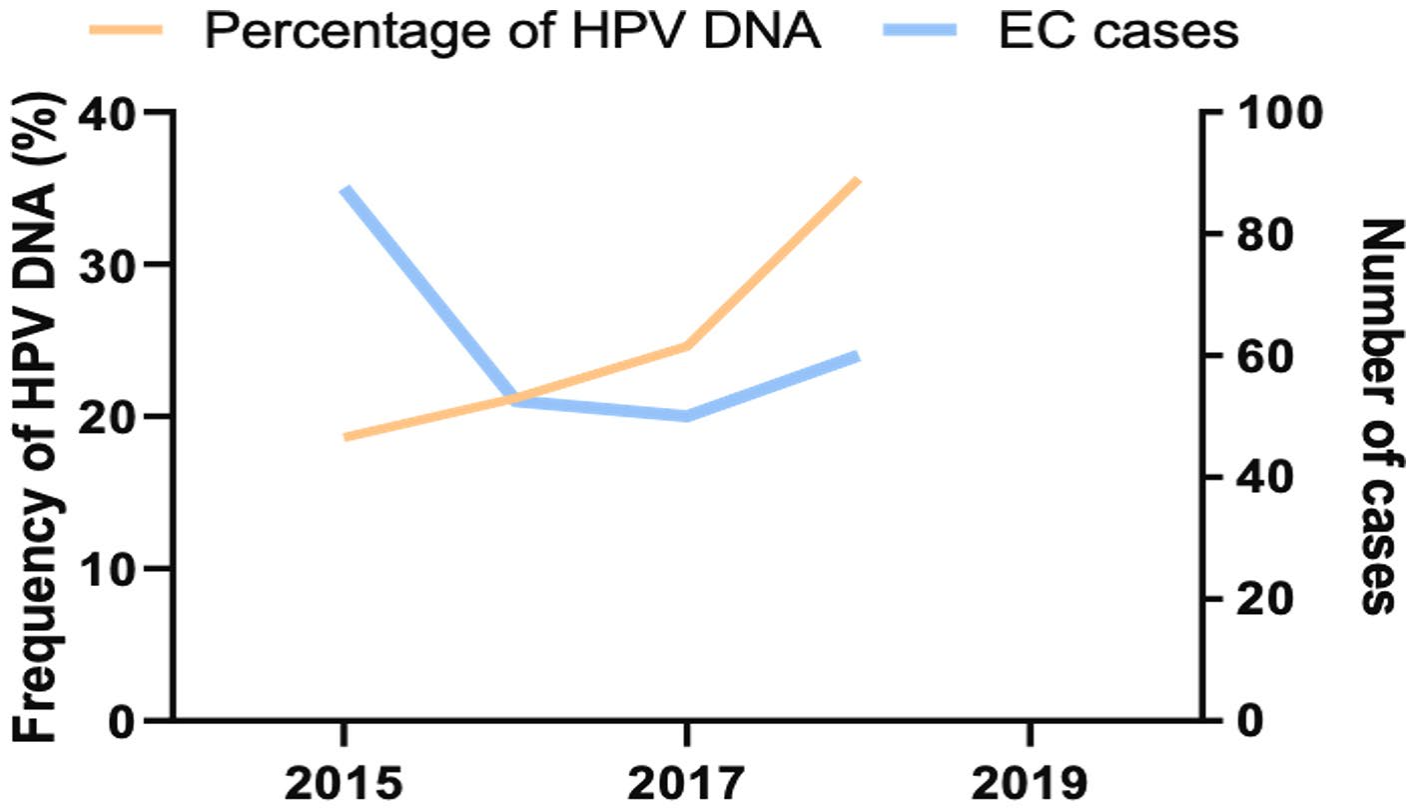
Prevalent trends for HPV-infected ECs in northeastern Thailand 2015–2018. The number of all HPV DNA-positive EC (orange) was indicated by the left y axis, while prevalent ECs (blue) were illustrated by the right y axis.

It has been reported that the occurrence of EC related to HPV infection is a favorable upward trend in the whole world, including Southeast Asia (Morgan et al., 2022). Then, we retrieved the frequency and temporal trend of HPV infection in EC tissue samples by assessing the presence of viral DNA over the collected year. The inclusive frequency of HPV-related ECs was 45.0% (Table 1). During the collection period, we observed favorable upward trends in HPV-attributable DNA frequency rates in registered EC tissue samples. The annual percentage of HPV DNA-positive cases was 18.6% in 2015, 21.2% in 2016, 24.6% in 2017, and 35.6% in 2018 (Figure 3). Altogether, our analysis provided additional information for an increasing trend of HPV-related ECs over time.

### 3.5. Meta-analysis indicated infection with hrHPV might play a crucial role in ECs as an etiological agent

**Figure 4:**
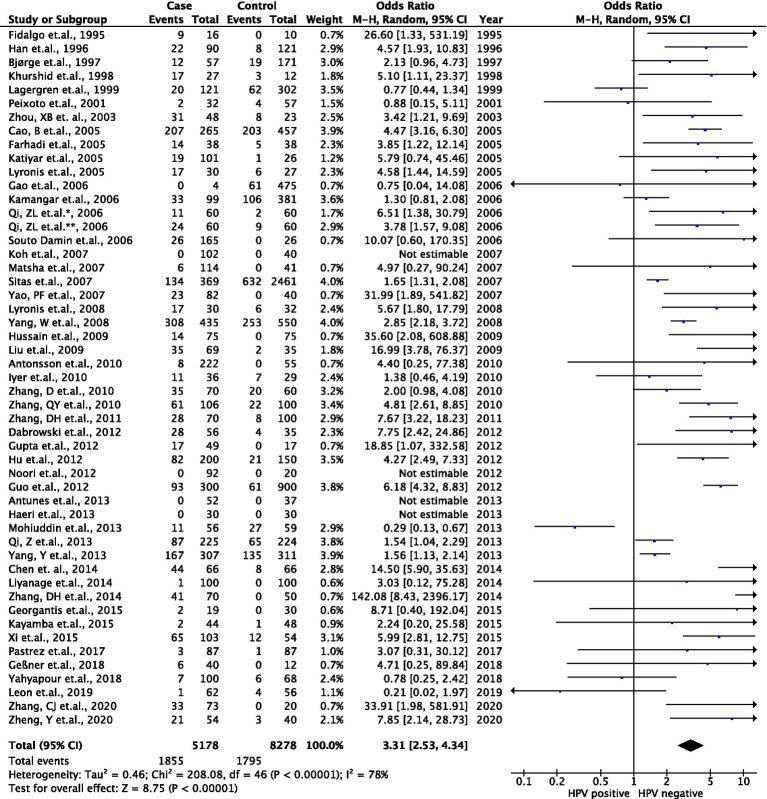
Association between HPV infection and EC risk in the meta-data analysis. Forest plots represented the pool odds ratios (ORs) and 95% CIs for the association between HPV infection and EC risk in each registered case–control study.

Although our recent results mentioned the association between HPV infection and the increasing risk of ECs, the evidence for this correlation is capricious in previous studies. Therefore, a recent meta-analysis was performed to clarify such a relation and verified our findings. When searching the four main public databases, a total of 51 case–control studies were selected as candidates in a recent meta-analysis (Supplementary Figure S1). Of such studies, 5,178 EC patient cases and 8,278 control participants were analyzed from 1995 to 2020. Among the registered reports, a total of 33 studies were held in the Asia continent, followed by seven from Europe, five from Africa, four from America, and two from Oceania. Interestingly, various techniques for HPV detection could be mentioned, including 34 studies that used PCR, 11 reports for antibodies (Ab), ELISA, *in situ* hybridization (ISH), and immunohistochemistry (IHC), one study for cytology, and five studies that used combination approaches. The type of biological specimens was also stated, consisting of 41 studies that collected formalin-fixed paraffin-embedded tissue (FFPE), seven reports for blood/plasma/serum, two studies for fresh frozen (FF) tissue /biopsies, and one exfoliated cell. The mean NOS score was 5.5 (Supplementary Table S1). Furthermore, the frequency of HPV DNA in ECs from enrolled studies was also described in the context of high or low EC risk (Supplementary Table S2). As shown in Figure 4, a total of 51 case–control studies were included and investigated the prevalence of HPV infection, including 5,178 EC patient cases and 8,278 control participants. The pooled prevalence of HPV in EC was 35.8% (1,855/5,178) while the rate of HPV infection was 21.9% (1,795/8,278) in the control arm. Using the random effect model (*I*^2^ = 78%; *p* < 0.001), the analytic result indicated that a significantly increased EC risk was associated with HPV infection (ORs, 3.3; 95% CI, 2.5–4.3; *p* < 0.001).

Since heterogeneity was observed in the result, we performed subgroup analyses and meta-regression on several main characteristics of registered studies, including geographic region, detection strategy, type of specimen, and risk of ECs in such countries. The presence of HPV DNA and the risk of ECs were positively correlated and consistently associated with all subgroups. Though some assessments were statistically significant, it was thought-provoking that the log ORs with 95% CIs for the relation between HPV infection and EC from different studied geographic regions were 1.29 (0.89–1.70) in Asia, 1.26 (0.27–2.24) in Europe, 0.50 (0.16–0.83) in Africa, 0.62 (−0.62–1.85) in America, and 1.32 (−1.04–3.67) in Oceania. Interestingly, we also perceived this association from the distinctive detection strategies that were 1.46 (1.14–1.77), 0.73 (0.01–1.44), and 0.83 (−1.61–3.27) in the detection of viral nucleic acid, viral protein, and combined markers, respectively, while we were unable to estimate the detection using the cytological assay. Furthermore, the log OR with 95% CI for the link between HPV infection and EC from different sample types was 1.41 (1.12–1.70) in the FFPE sample, 0.43 (−0.48–1.34) in the blood/plasma/serum sample, and 0.38 (−22.39–23.15) in the FF/biopsy sample, and not estimated in the sample obtained from exfoliated cells. Our analysis of this correlation from the variation in EC risk presented the log ORs with 95% CIs that were 1.22 (0.84–1.61) and 1.06 (0.48–1.63) in high EC risk countries and low EC risk countries, respectively. Additionally, the meta-regression unveiled that the characteristics of the enrolled studies did not significantly affect the analytic results (*p* > 0.05), except for the type of biological samples (*p* = 0.015), and moderately rationalized the source of heterogeneity. Our analyses in all subgroups and meta-regression are illustrated in Table 5. Subsequently, the sensitivity of a recent meta-analysis (Egger’s test, *p* < 0.05) and publication bias (Supplementary Figure S2) were monitored, which involved the existing risk of HPV infection in EC populations. By the leave-one-out analysis, we realized that the ORs were constantly steadily omitting any individual reports (data not shown), which statistically tested the dependability of the current meta-analysis. Taken together, our inquiries implied that HPV infection was evident in approximately 20% of totally different EC cases throughout the global studies.

**Table 5:** Evaluation of the involvement of the HPV infection on EC risk in the subgroup context.

Characterization	Number of studies	Log pooled OR (95% CI)^d^	*p*-value	*I*^2^ (%)	*p*-value for heterogeneity	Meta-regression
Overall	51	1.2 (0.89–1.49)	<0.001	81.8	<0.001	–
Geographic region						
Asia	33	1.29 (0.89–1.70)	<0.001	84.9	<0.001	0.176
Europe	7	1.26 (0.27–2.24)	0.02	72.6	<0.001	
Africa	5	0.50 (0.16–0.83)	0.02	0	0.36	
America	4	0.62 (−0.62–1.85)	0.20	0	0.56	
Oceania	2	1.32 (−1.04–3.67)	0.09	0	0.865	
Detection strategy^**a**^						
Nucleic acid, e.g., PCR	34	1.46 (1.14–1.77)	<0.001	65.7	<0.001	0.474
Protein, e.g., Ab, ELISA, ISH, and IHC	11	0.73 (0.01–1.44)	0.05	90.0	<0.001	
Cell, i.e., cytology	1	NE	NE	NE	NE	
Combined technique	5	0.83 (−1.61–3.27)	0.04	61.5	0.051	
Type of specimen^**b**^						
PE	41	1.41 (1.12–1.70)	<0.001	66.4	<0.001	0.015
Blood/plasma/serum	7	0.43 (−0.48–1.34)	0.29	90.7	<0.001	
FF/biopsies	2	0.38 (−22.39–23.15)	0.87	87.3	0.005	
Exfoliated cells	1	NE	NE	NE	NE	
Risk of ECs^**c**^						
High	37	1.22 (0.84–1.61)	<0.001	86.1	<0.001	0.309
Low-medium	14	1.06 (0.48–1.63)	<0.001	51.4	0.007	

OR, odd ratio; CI, confidence interval; HPVs, human papillomaviruses; EC, esophageal cancers; NE, did not estimated.

a PCR, polymerase chain reaction; Ab, antibodies; ISH, *in situ* hybridization; IHC, immunohistochemistry; ELISA, enzyme-linked immunosorbent assay; WB, Western blotting.

b PE, formalin-fixed paraffin-embedded tissue; FF, Fresh frozen tissue.

c Rajendra et al. (2020), Ludmir et al. (2015), and Kunzmann et al. (2017).

d When *I*^2^  < 50% or *p*-value for heterogeneity > 0.10, the fixed effect model was applied.

### 3.6. HPV infection might not affect the prognosis of EC patients

To determine the involvement of HPV in the prognosis of EC, the association between HPV infection and the prognostic characteristics of EC patients was consequently performed. As shown in Table 6, OR_adj._ with 95% CI of the three potential risk factors, including smoking, history of GERD, and HPV infection, showed a similar trend in the two different histopathological gradings (poor differentiation vs. moderate/well differentiation) of EC cases. In addition, in the analysis of the TNM stage, we observed favorable downward trends of these risk factors in the EC cases. Collectively, these results indicated that the status of HPV might not affect the survival of EC patients for better or worst prognosis context.

**Table 6:** Relationship of the potential risk factors and the prognostic features for ECs.

Characteristics	Histopathological grade (*N* = 96)		OR_adj_ (95% CI)	*p*-value^a^	TNM stage (*N* = 100)		OR_adj_ (95% CI)	*p*-value^a^
	Poor diff.; *n* (%)	Moderate/well diff.; *n* (%)			Stage I, II; *n* (%)	Stage III, IV; *n* (%)		
Smoking								
Yes	48 (72.7)	21 (70.0)	1.1 (0.4–2.9)	0.837	7 (77.8)	65 (71.4)	0.9 (0.2–4.6)	0.872
No	18 (27.3)	9 (30.0)	1.0 (reference)		2 (22.2)	26 (28.6)	1.0 (reference)	
History of GERD								
Yes	53 (80.3)	23 (76.7)	1.3 (0.4–3.7)	0.664	9 (100.0)	70 (76.9)	0.0 (0.0)	0.998
No	13 (19.7)	7 (23.3)	1.0 (reference)		0 (0.0)	21 (23.1)	1.0 (reference)	
HPVs status								
Positive	32 (48.5)	11 (36.7)	1.6 (0.7–4.0)	0.664	5 (55.6)	40 (44.0)	0.6 (0.1–2.4)	0.470
Negative	34 (51.5)	19 (63.3)	1.0 (reference)		4 (44.4)	51 (56.0)	1.0 (reference)	

Poor diff., poor differentiation; Moderate/well diff., moderate or well differentiation; OR_adj_, adjusted odd ratio; CI, confidence interval; GERD, gastroesophageal reflux disease; HPVs, human papillomaviruses.

a *p*-value was obtained from a multiple logistic regression model with all conditional adjusted variables.

## 4. Discussions

Currently, the number of pieces of epidemiological evidence that indicated an association between hrHPV and the risk of EC has increased. However, the involvement of such a virus in EC pathogenesis is still debated. The variation in its geographical incidence, which was affected by exposure to dietary, cultural, and environmental factors, and any specific factors, is revealed to be one of the reasons that might be described as potential factors. To clarify the carcinogenic roles of hrHPV in EC pathogenesis, we, therefore, conducted a retrospective case–control study and meta-analyses of public databases to describe the relationship between hrHPV infection and the EC risk among Thai patients who lived in the northeastern part. As our hypothesis, the results indicated whether HPV infection, synergized with the history of GERD in patients, was statistically associated with an increased risk of EC, supporting that HPV may, together with the history of GERD, act as a significant etiological risk factor for EC.

Human papillomaviruses (HPVs) are double-stranded circular DNA with small, non-enveloped species-specific viruses, belonging to the *Papillomaviridae* family compartment. A total of 12 alpha-HPV genotypes from alpha-HPVs (e.g., HPV16, HPV18, and HPV33) are etiological agents of several types of cancer in humans, not only in the anogenital tract but also in a subset of the origin of the head and neck, particularly the oropharynx and oral cavity (Gheit, 2019). According to previous epidemiologic studies, infection with HPV16 and HPV18 was certified in most cases of cervical cancer, accounting for 50% and 20%, respectively (Garland, 2002; Wentzensen et al., 2009; de Sanjose et al., 2010). The malignancies that arose in the head and neck subgroups were mostly found in the base of the tongue, tonsils, and oropharynx (D’Souza et al., 2007; Gillison et al., 2012; Phusingha et al., 2017). The rate of HPV infection in oropharyngeal cancer was 11.5% for any viral genotype and 7.3% for hrHPV among men, while the prevalence of any type of HPV infection and high-risk type was 3.2% and 1.4%, respectively, among women (Sonawane et al., 2017). HPV16 is the most potent oncogenic genotype of the hrHPVs subgroup.

Infections with HPV have been implied as the oncogenic driver, implicated in the pathogenesis of ECs, particularly in ESCC by the accumulated evidence (Stoner and Gupta, 2001; Eslick, 2010; El-Zimaity et al., 2018). Recently, the correlated HPV16 and HPV18 genotypes were the most studied. However, the association between HPV infection and the increased risk of ECs has also been supposed unsettled. The previous larger scale study with go steady more than 5 years ago by using meta-data analysis indicated the range of HPV frequency between 0% and 100% with the rate of the difference in the HPV subtypes, concurring to the geographic region and the type of analytic study (Li et al., 2014; Petrick et al., 2014; Ludmir et al., 2015). As expected and consistent with previous reports, the Asian continent was the geographical area that found the highest prevalence of HPV-infected ECs. Of these, HPV16 showed the highest prevalence compared to HPV18 in the ESCC subtype. However, the report of the diverse population of both observational and case–control infection studies indicated that infections with HPV16 and HPV18 were more common in EC patients from the eastern part of the world (Europe, the Americas, and Africa) and had an ESCC connection compared to healthy subjects. Despite the presence of variations across the studied types, most of the significant association was observed with less variation in the case–control model (Asian vs. non-Asian subjects). Similarly, in the multivariate regression analysis, the ORs indicated the statistical correlation between smoking, alcohol consumption, history of GERD, and FHC but were not observed in variation of age and gender of enrolled subjects. The associated results from the analysis of head and neck cancers were contrary (Gillison et al., 2008). However, the p16^INK4a^ protein was the potentially promising biological marker for HPV-related malignancies and presented more precise corroboration with DNA testing using a PCR- or immunohistochemistry (IHC)-based technique for HPV diagnosis (at least in oropharyngeal carcinoma). Nevertheless, most studies targeting HPV DNA without p16^INK4a^ evaluation could not be a gold standard, therefore defining the association between HPV infection and increased risk of EC in this recent meta-analysis.

It is extraordinary that the genomic landscape of ECs has been described by The Cancer Genome Atlas Research Network since 2017 (Kim et al., 2017). They illustrated three distinct existences of molecular subclassification in ESCC. Although aberrations in the Nrf2 pathway were the hallmark of the ESCC1 subtype, higher mutation rates in NOTCH1 or ZNF750 were the genomic characterization of ESCC2. Furthermore, this report did not indicate any evidence of cell cycle dysregulation and p53 mutations in p53 at the genomic level for ESCC3. However, the expression of p53 did not declare an association with HPV-linked ESCC, as the mutations of the p53 gene were measurable in both ESCC with and without HPV infection, implying the reciprocally inclusive consequences between p53 mutations and HPV infections. Thus, the important role of environmental carcinogens could be signified in the oncogenesis of ESCCs (Chang et al., 1994).

The overexpression of p16^INK4a^ has been described as a surrogate marker for hrHPV-associated cancer of the oropharynx through the eighth edition of the American Joint Committee on Cancer Staging Manual (Lydiatt et al., 2017). However, the relationship between ECs and HPV infection is still debated. It has been proposed that anatomical and environmental causes might be allied to underlying reasons. The esophageal epithelium and squamous cell malignancy may not be more affected by the high risks of sexual behavior than EAC (Wong et al., 2018). In Asianic populations, dietary factors, including poor status nutrition, high temperature of drinking beverages, and low uptake of fruits and vegetables, might be regarded as risk rationales (Tran et al., 2005; Li et al., 2011). On the contrary, smoking and alcohol drinking were major factors in the Westerner risk (Engel et al., 2003; Freedman et al., 2007). Despite this, the raise of ECs could be augmented by HPV-positive status, intensely in patients with a history of smoking and alcohol consumption (at least in China; Qi et al., 2013) but not transpire in the countries of the Western parts as demonstrated by Georgantis et al. (2015). In the recent study, we also revealed that there is no associated evidence of HPV DNA status and prognosis in EC patients, suggesting that no significant effect of HPV infection on patient survival appears to be consistent with the previous few studies that informed the conclusion results (Petrelli et al., 2021). Similarly, the effects of HPV infection on Barrett’s metaplasia and EAC were still debated, and no strong evidence could be determined in the systematic review and meta-analysis (Rajendra et al., 2020).

In harmony with the previous reports, we defined several limitations of the current analysis with a certain association. Most studies, which represent 50%, were derived from Asian people with differences in genomic landscape, mental origin, and daily life. Critically, variations in the evaluation essay, line sensitivities, and types of specimens used could have primarily shaped the summarized data. As we mentioned, FFPE tissues were supposed to be specimens of suggestive deprivation compared with FF or biopsies. Furthermore, the most enrolled studies ratified the existence of HPVs based only on PCR, despite the p16^INK4a^ IHC, which was the highly sensitive standard approach to the diagnosis of HPV-associated malignancies. Consequently, there were few studies conducted with the case–control analysis, describing the risk association based on the crude information and the weight of influenced variables (e.g., smoking status and alcohol history). Additionally, the potential of hrHPVs was emphasized in squamous histology.

An aforementioned study of meta-studies indicated a positive correlation of HPV in EC patients only in the Asian population (Yong et al., 2013). However, our results overtalked this analysis and were also harmoniously observed through the moderately significant association of ECs with HPV-positive in altered geographical prevalence, especially in both Asian and Westerners, and what about our data? GERD history, smoking, and alcohol consumption seem to play a concomitant or synergistic role with HPV infection.

Despite the recent study that clarified and supported the involvement of hrHPV in EC oncogenesis, some critical points must be elucidated. Remarkably, the differences in study design and viral detection approach (e.g., the sensitivity of method) affected the quality of the OR results (i.e., adjusted and pooled OR) as well as the prevalence of HPV in EC tissue samples, respectively. We were able to analyze the clinico-demographic data only in a structured questionnaire, and the other environmental risk factors might be lost from the original patient’s life. Importantly, some studies of HPV infection included inadequate sample sizes, affecting our analysis and difficult to summarize. Although more largely epidemiological studies are necessary to show the strength of this correlation, recent data have shed light on therapeutic purposes and preventive epidemiology of ECs. Furthermore, applicable studies adhere to the established procedural standards that were adhered to by other tumor articles and should be integrated into ECs to provide adequate knowledge on HPV-driven EC pathogenesis. Furthermore, the precision-treated responses and effective-prevented vaccines of HPV-associated ECs could be invented in future research.

## 5. Conclusion

We specify the recent epidemiologic evidence in a validation of the distributed HPV which might be statistically associated with an increased risk of EC and seems to be enhanced by GERD history, suggesting that HPV may, together with GERD history, act as a significant etiological risk factor of ECs. However, additional high-quality studies with larger sample sizes are needed to further verify the link between HPV and EC.

## Data availability statement

The raw data supporting the conclusions of this article will be made available by the authors, without undue reservation.

## Ethics statement

The studies involving human participants were reviewed and approved by the Khon Kaen University Ethics Committee for Human Research. The patients/participants provided their written informed consent to participate in this study.

## Author contributions

AB, CP, TE, and TN designed and guided the literature. AB, PT, WW, and AP executed data acquisition, analysis, and interpretation. AB, CP, and TE wrote the review and revised the manuscript. AU, PS, PU, and TN administrated, technically, or materially supported the study. CP and TN were the study’s supervisors. All authors contributed to the article and approved the submitted version.

## Funding

This study was funded by a scholarship under the Post-Doctoral Training Program from Khon Kaen University, Thailand (grant no. PD2565-08) to AB. This study was supported in part by Research and Graduate Studies, Khon Kaen University, Thailand (RP65-8-001) to CP.

## Conflict of interest

AU was employed by Ward Medic Ltd., Part., Bangkok, Thailand.

The remaining authors declare that the research was conducted in the absence of any commercial or financial relationships that could be construed as a potential conflict of interest.

## Publisher’s note

All claims expressed in this article are solely those of the authors and do not necessarily represent those of their affiliated organizations, or those of the publisher, the editors and the reviewers. Any product that may be evaluated in this article, or claim that may be made by its manufacturer, is not guaranteed or endorsed by the publisher.

## Footnotes

^1^ https://training.cochrane.org/online-learning/core-software/revman/revman-5-download
