# Supplementary material for: Epidemiological evidence and association of human papillomavirus with esophageal cancer in northeastern Thailand: a case–control study
Source: Front Microbiol. 2023 Apr 27;14:1146322. doi: 10.3389/fmicb.2023.1146322 (PMC10172481; doi:10.3389/fmicb.2023.1146322)
Supplement: Supplementary file 1 [file Data_Sheet_1.docx]

Supplementary Material

## Supplementary Figures and Tables

**Supplementary Figure S1.** Flow diagram of enrolled studies.

**
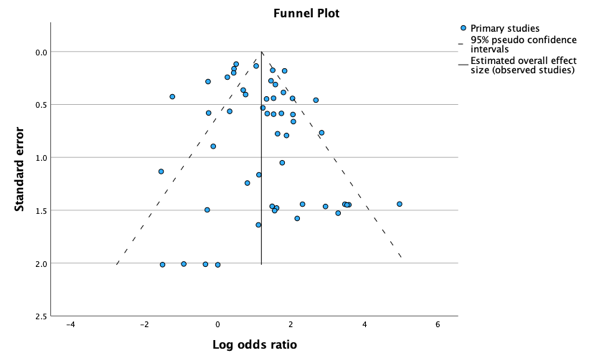
**

**Supplementary Figure S2.** Analysis of publication bias for studies in the meta-analysis. A funnel plot was performed to evaluate the bias in the included publications based on Begg’s random meta-analysis.

**Supplementary Table S1. The depiction of the reports included in a meta-analysis**

| **Year** | **Autor** | **Study region** | **Number of samples, n** | | | | **Detection strategy** | **Type of sample**  **(Cases/controls)** | **NOS** |
| --- | --- | --- | --- | --- | --- | --- | --- | --- | --- |
|  |  |  | **Cases** | | **Controls** | |  |  |  |
|  |  |  | **HPV (+)** | **HPV (-)** | **HPV (+)** | **HPV (-)** |  |  |  |
| 1995 | Fidalgo et.al. | Portugal | 9 | 7 | 0 | 10 | PCR | PE/PE | 5 |
| 1996 | Han et.al. | China | 22 | 68 | 8 | 113 | ELISA | Serum | 5 |
| 1997 | Bjørge et.al. | Norway | 12 | 45 | 19 | 152 | Circulated Ab | Serum | 5 |
| 1998 | Khurshid et.al. | Japan | 17 | 10 | 3 | 9 | PCR | PE/PE | 5 |
| 1999 | Lagergren et.al. | Sweden | 20 | 101 | 62 | 240 | Circulated Ab | Serum | 5 |
| 2001 | Peixoto et.al. | China | 2 | 30 | 4 | 53 | Cytology | Exfoliated cells | 5 |
| 2003 | Zhou, XB et. al. | China | 31 | 17 | 8 | 15 | PCR/ISH | PE/PE | 5 |
| 2005 | Cao, B et.al. | China | 207 | 58 | 203 | 254 | PCR | PE/PE | 6 |
| 2005 | Farhadi et.al. | Iran | 14 | 24 | 5 | 33 | PCR | PE/PE | 5 |
| 2005 | Katiyar et.al. | India | 19 | 82 | 1 | 25 | PCR | PE/PE | 5 |
| 2005 | Lyronis et.al. | Greece | 17 | 13 | 6 | 21 | PCR | PE/PE | 5 |
| 2006 | Gao et.al. | China | 0 | 4 | 61 | 414 | PCR | PE/PE | 5 |
| 2006 | Qi, ZL et.al. | China | 11 | 49 | 2 | 58 | IHC | PE/PE | 5 |
| 2006 | Qi, ZL et.al. | China | 24 | 36 | 9 | 51 | ISH | PE/PE | 5 |
| 2006 | Kamangar et.al. | China | 33 | 66 | 106 | 275 | Circulated Ab | Serum | 6 |
| 2006 | Souto Damin et.al. | Brazil | 26 | 139 | 0 | 26 | PCR | PE/PE | 6 |
| 2007 | Yao, PF et.al. | China | 23 | 59 | 0 | 40 | IHC/ISH | PE/PE | 5 |
| 2007 | Koh et.al. | Korea | 0 | 102 | 0 | 40 | PCR | PE/PE | 5 |
| 2007 | Matsha et.al. | South Africa | 6 | 108 | 0 | 41 | PCR | PE/PE | 5 |
| 2007 | Sitas et.al. | South Africa | 134 | 235 | 632 | 1829 | ELISA | Serum | 7 |
| 2008 | Lyronis et.al. | Greece | 17 | 13 | 6 | 26 | PCR | PE/PE | 5 |
| 2008 | Yang, W et.al. | China | 308 | 127 | 253 | 297 | PCR | PE/PE | 7 |
| 2009 | Hussain et.al. | India | 14 | 61 | 0 | 75 | PCR/WB | PE/PE | 5 |
| 2009 | Liu et.al. | China | 35 | 34 | 2 | 33 | PCR | PE/PE | 5 |
| 2010 | Zhang, D et.al. | China | 35 | 35 | 20 | 40 | PCR | PE/PE | 5 |
| 2010 | Zhang, QY et.al. | China | 61 | 45 | 22 | 78 | PCR | PE/PE | 6 |
| 2010 | Antonsson et.al. | Australia | 8 | 214 | 0 | 55 | PCR | PE/PE | 6 |
| 2010 | Iyer et.al. | USA | 11 | 25 | 7 | 22 | PCR | PE/PE | 6 |
| 2011 | Zhang, DH et.al. | China | 28 | 42 | 8 | 92 | PCR | PE/PE | 6 |
| 2012 | Dabrowski et.al. | Poland | 28 | 28 | 4 | 31 | PCR | Biopsy | 5 |
| 2012 | Guo et.al. | China | 93 | 207 | 61 | 839 | PCR | PE/PE | 6 |
| 2012 | Gupta et.al. | India | 17 | 32 | 0 | 17 | PCR | PE/PE | 5 |

**Supplementary Table S1.** continued

| **Year** | **Autor** | **Study region** | **Sample size** | | | | **Detection strategy^a^** | **Type of sample**  **(Cases/controls)^b^** | **NOS** |
| --- | --- | --- | --- | --- | --- | --- | --- | --- | --- |
|  |  |  | **Cases** | | **Controls** | |  |  |  |
|  |  |  | **HPV (+)** | **HPV (-)** | **HPV (+)** | **HPV (-)** |  |  |  |
| 2012 | Hu et.al. | Northwest China | 82 | 118 | 21 | 129 | PCR | PE/PE | 6 |
| 2012 | Noori et.al. | Iran | 0 | 92 | 0 | 20 | PCR | PE/PE | 5 |
| 2013 | Yang, Y et.al. | China | 167 | 140 | 135 | 176 | Circulated Ab | Blood | 7 |
| 2013 | Antunes et.al. | Brazil | 0 | 52 | 0 | 37 | PCR | PE/PE | 5 |
| 2013 | Haeri et.al. | India | 0 | 30 | 0 | 30 | PCR | PE/PE | 5 |
| 2013 | Mohiuddin et.al. | India | 11 | 45 | 27 | 32 | PCR | PE/PE | 5 |
| 2013 | Qi, Z et.al. | China | 87 | 138 | 65 | 159 | ELISA | Plasma | 6 |
| 2014 | Zhang, DH et.al. | China | 41 | 29 | 0 | 50 | PCR | PE/PE | 7 |
| 2014 | Chen et. al. | China | 44 | 22 | 8 | 58 | PCR | PE/PE | 5 |
| 2014 | Liyanage et.al. | Australia | 1 | 99 | 0 | 100 | PCR | PE/PE | 6 |
| 2015 | Georgantis et.al. | Greece | 2 | 17 | 0 | 30 | PCR | PE/PE | 5 |
| 2015 | Kayamba et.al. | Zambia | 2 | 42 | 1 | 47 | PCR | PE/PE | 5 |
| 2015 | Xi et.al. | China | 65 | 38 | 12 | 42 | Tissue Ab | PE/PE | 8 |
| 2017 | Pastrez et.al. | Brazil | 3 | 84 | 1 | 86 | PCR | PE/PE | 5 |
| 2018 | Geßner et.al. | Malawi | 6 | 34 | 0 | 12 | PCR/ISH/p16^INK4a^ | PE/PE | 5 |
| 2018 | Yahyapour et.al. | Iran | 7 | 93 | 6 | 62 | PCR | PE/PE | 6 |
| 2019 | Leon et.al. | Ethiopia | 1 | 61 | 4 | 52 | PCR/Luminex | FF | 5 |
| 2020 | Zhang, CJ et.al. | China | 33 | 40 | 0 | 20 | ISH | PE/PE | 7 |
| 2020 | Zheng, Y et.al. | China | 21 | 33 | 3 | 37 | PCR | PE/PE | 6 |

^a^ PCR, polymerase chain reaction; Ab, antibodies; ISH, *in situ* hybridization; IHC, immunohistochemistry; ELISA, enzyme-linked immunosorbent assay; WB, Western blotting.

^b^PE, formalin-fixed paraffin-embedded tissue; FF, Fresh frozen tissue.

**Supplementary Table S2. Frequency of HPV DNA in esophageal cancer from Case-Control study according to geographic region**

| **Geographic region** | **Risk of ECs^a^** | **Number of samples tested** | | **Number of HPV-positive samples** | | **Number of studies** |
| --- | --- | --- | --- | --- | --- | --- |
|  |  | **Cases** | **Controls** | **Cases** | **Controls** |  |
| **Asia** |  |  |  |  |  |  |
| China | High | 2,888 | 4,334 | 1,453 | 1,011 | 23 |
| India | High | 311 | 207 | 61 | 28 | 5 |
| Iran | High | 230 | 126 | 21 | 11 | 3 |
| Japan | High | 27 | 12 | 17 | 3 | 1 |
| Korea | Low-medium | 102 | 40 | 0 | 0 | 1 |
| **Oceania** |  |  |  |  |  |  |
| Australia | Low-medium | 322 | 155 | 9 | 0 | 2 |
| **Europe** |  |  |  |  |  |  |
| Greece | Low-medium | 79 | 89 | 36 | 12 | 3 |
| Portugal | Low-medium | 16 | 10 | 9 | 0 | 1 |
| Norway | Low-medium | 57 | 171 | 12 | 19 | 1 |
| Sweden | Low-medium | 121 | 302 | 20 | 62 | 1 |
| Poland | Low-medium | 56 | 35 | 28 | 4 | 1 |
| **America** |  |  |  |  |  |  |
| Brazil | Low-medium | 304 | 150 | 29 | 1 | 3 |
| USA | Low-medium | 36 | 29 | 11 | 7 | 1 |
| **Africa** |  |  |  |  |  |  |
| South Africa | High | 483 | 2502 | 140 | 632 | 2 |
| Zambia | High | 44 | 48 | 2 | 1 | 1 |
| Malawi | High | 40 | 12 | 6 | 0 | 1 |
| Ethiopia | High | 62 | 56 | 1 | 4 | 1 |

ECs, esophageal cancers; HPV, human papillomavirus

^a^Refs: (12,15,20)
